# High Resolution Covid Program. Purposely a case

**DOI:** 10.1192/j.eurpsy.2022.1382

**Published:** 2022-09-01

**Authors:** N. Ogando Portilla, S.M. Bañón González, M. Martínez Cortés, M. Agudo Urbanos, R. Gutiérrez Labrador

**Affiliations:** 1 Hospital Infanta Sofía, Psychiatry, San Sebastian de los Reyes (Madrid), Spain; 2 Hospital General Universitario de Alicante, Psychiatry, Alicante, Spain; 3 Hospital Universitario La Princesa, Psychiatry, madrid, Spain

**Keywords:** High resolution program, covid

## Abstract

**Introduction:**

The covid pandemic has become a unique phenomenon in world history with great impact on mental health.

**Objectives:**

A great growth of anxious depressive pathology in relation to the Covid situation has appeared with the need to increase the psychiatric approach in the general population

**Methods:**

A 58-year-old woman with no personal medical story of interest is referred to the high-resolution Covid program due to severe depressive symptoms: intense apathy, abulia, anhedonia, weight loss, insomnia and important social distancing after the beginning of the confinement due to the Covid Pandemic. 4 psychotherapy sessions are performed, with a maximum duration of 45 minutes. It is necessary to add antidepressant medication with sertraline up to 100mg to improve psychotherapeutic work.

**Results:**

A complete recovery of symptoms is achieved even their severity with normalization of daily life.

**Conclusions:**

Small psychotherapeutic interventions have been shown, even with critically ill patients, to be very effective in helping patients regain their baseline status.

**Disclosure:**

No significant relationships.

